# Supporting patient involvement in health service research: development of a methodological guidance: Marie Dauvrin

**DOI:** 10.1093/eurpub/ckac130.162

**Published:** 2022-10-25

**Authors:** M Dauvrin, L Kohn, I Cleemput

**Affiliations:** Belgian Health Care Knowledge Centre, Brussels, Belgium

## Abstract

**Background:**

The Belgian Health Care Knowledge Centre (KCE) issued a Position Paper in 2019 regarding the Patient Involvement (PI) in its researches as stakeholders. After previously investigating the organizational culture of the KCE, a key element for a successful implementation of PI was providing methodological guidance to the researchers.

**Objectives:**

The objective of this study was to develop a methodological guidance for implementing PI into the practices of researchers at KCE.

**Methods:**

In order to build a practical and effective process note, reflecting the needs and points of attention of all involved participants, different methods were combined: a literature review, workshops with the umbrellas of patient associations, patients and patient representatives, Delphi survey and a pilot project involving patients as research partners.

**Results:**

The resulting guidance identified 5 prerequisites and conditions for implementation of patient involvement at the organisational level. The guidance also focused on how to involve patients (which patients, how, when, what for) and included general recommendations to researchers during the collaboration (communication, relational aspects, animation techniques, specific needs of patients). Recommendations for the reporting and evaluation of PI were also formulated. Alongside the guidance for researchers, supports for patients were also developed and validated by the patient associations.

**Conclusions:**

The KCE guidance for PI in research could inspire other agencies willing to implement PI in their practices. Implementation will therefore require additional human and financial resources.

**Key messages:**

- Patient involvement into resarch needs methodological guidance to be successful.
- Cocreated guidance for patient involvement could help other researchers.

